# Higher plant-derived nitrate intake is associated with lower odds of frailty in a cross-sectional study of community-dwelling older women

**DOI:** 10.1007/s00394-024-03412-z

**Published:** 2024-05-18

**Authors:** Eleanor Hayes, Elsa Dent, Oliver M. Shannon, Lie Zhou Zhong, Trent Bozanich, Lauren C. Blekkenhorst, Kun Zhu, Catherine P. Bondonno, Mario Siervo, Emiel O. Hoogendijk, Jonathan M. Hodgson, Richard L. Prince, Joshua R. Lewis, Marc Sim

**Affiliations:** 1 Faculty of Health and Life Sciences, Northumbria University, Newcastle upon Tyne, UK; 2 Research Centre for Public Health, Equity and Human Flourishing, Torrens University Australia, Adelaide, South Australia Australia; 3 Human Nutrition and Exercise Research Centre, Population Health Sciences Institute, Newcastle University, Newcastle upon Tyne, UK; 4 Nutrition and Health Innovation Research Institute, School of Health and Medical Sciences, Edith Cowan University, Perth, WA Australia; 5 Medical School, The University of Western Australia, Perth, WA Australia; 6 Deparment of Endocrinology and Diabetes, Sir Charles Gairdner Hospital, Perth, WA Australia; 7 School of Public Health, Curtin University, Perth, WA Australia; 8 Department of Epidemiology and Data Science, VU University Medical Center, Amsterdam UMC, Amsterdam, Netherlands; 9 Ageing and Later Life Research Program, Amsterdam Public Health Research Institute, Amsterdam UMC, Amsterdam, Netherlands

**Keywords:** Nitric oxide, Womens health, Plant-foods, Frailty index, Cumulative deficits

## Abstract

**Purpose:**

Dietary nitrate intake is inversely related to numerous contributors towards frailty, including cardiovascular disease and poor physical function. Whether these findings extend to frailty remain unknown. We investigated if habitual nitrate intake, derived from plants or animal-based foods, was cross-sectionally associated with frailty in women.

**Methods:**

Community-dwelling older Australian women (*n* = 1390, mean age 75.1 ± 2.7 years) completed a validated semi-quantitative food frequency questionnaire (FFQ). Nitrate concentrations in food were obtained from international nitrate databases. We adopted the Rockwood frailty index (FI) of cumulative deficits comprising 33 variables across multiple health domains (scored 0 to 1), which predicts increased hospitalisation and mortality risk. A FI ≥ 0.25 indicated frailty. Cross-sectional associations between nitrate intake (total plant and animal nitrate, separately) and frailty were analysed using multivariable-adjusted logistic regression models (including lifestyle factors), as part of restricted cubic splines.

**Results:**

A non-linear inverse relationship was observed between total plant nitrate intake and frailty. Compared to women with the lowest plant nitrate intake (Quartile [Q]1), women with greater intakes in Q2 (OR 0.69 95%CI 0.56–0.84), Q3 (OR 0.67 95%CI 0.50–0.90) and Q4 (OR 0.66 95%CI 0.45–0.98) had lower odds for frailty. A nadir in the inverse association was observed once intakes reached ~ 64 mg/d (median Q2). No relationship was observed between total animal nitrate and frailty.

**Conclusion:**

Community-dwelling older women consuming low amounts of plant-derived nitrate were more likely to present with frailty. Consuming at least one daily serving (~ 75 g) of nitrate-rich green leafy vegetables may be beneficial in preventing frailty.

**Supplementary Information:**

The online version contains supplementary material available at 10.1007/s00394-024-03412-z.

## Introduction

Frailty is a clinical state involving declines across multiple physiological systems (e.g., sensory, neurological, cardiovascular, and musculoskeletal) which results in an increased vulnerability to adverse health outcomes and death [1–3]. Specifically, the Rockwood cumulative deficit model of frailty (frailty index, [FI]) comprises a reduced ability to perform activities of daily living due to losses in muscle strength and physical function, impaired cognitive function, and the presence of comorbidities such as vascular disease [4]. This makes the FI a multidimensional measure encompassing a wide range of health domains. Although the presence of frailty increases with age, progression of the condition is not linear but dynamic, and it may be possible to prevent or delay the onset of frailty via modulation of key risk factors for this condition [4].

Nutrition is an important modifiable risk factor for frailty. Elucidating the nutrients, foods or dietary patterns associated with frailty risk is essential for the development of appropriate dietary interventions, as well as public health messaging [5]. Several foods and dietary patterns have been identified in lowering frailty risk including diets with anti-inflammatory potential, diets high in protein and vitamin D intake, as well as the Mediterranean diet [6]. On the other hand, ultra-processed foods, including processed meats, have been linked with adverse effects on frailty and mortality due to these foods being energy dense, low in beneficial nutrients and phytochemicals, and being potentially pro-inflammatory [7].

Healthier dietary patterns linked with reduced frailty are often characterised by a greater consumption of fruits and vegetables [5]. There is growing evidence to suggest that the health benefits observed from a vegetable-rich diet may, at least in part, be explained by higher intake of inorganic dietary nitrate [8, 9]. Ingested dietary nitrate is metabolised to nitric oxide (NO), a pleiotropic signalling molecule shown to influence multifarious functions in the body, through the nitrate–nitrite–nitric oxide pathway [10, 11]. The increased bioavailability of NO has the potential to positively impact several components of frailty. For example, dietary nitrate supplementation has been reported to lower blood pressure, reverse vascular dysfunction, support cognitive function and lower risk of cardiovascular and neurodegenerative diseases in older adults [12]. Higher habitual dietary nitrate intake has also been associated with greater muscle strength (e.g., hand grip and knee extension strength), greater muscle power [13], and improved physical function (e.g., timed-up-and-go) [14, 15]. Such benefits may be mediated by enhanced skeletal muscle contractile properties, improved efficiency of mitochondrial respiration (reported by some [16] but not others [17]), and increased blood flow to active muscle [18, 19].

Given the potential for dietary nitrate intake to positively influence several of the aforementioned components of frailty, it is reasonable to expect that older adults with higher habitual nitrate intake would be less likely to present with frailty. However, this association is yet to be explored. Therefore, our aim was to determine if nitrate intake was cross-sectionally associated with a multi-dimensional measure of frailty, such as the FI, in community-dwelling older women. Finally, as it is currently unknown if nitrate from vegetables versus animal sources is equal in benefit/harm [12], we considered the impact of these two nitrate sources separately in our analysis.

## Method

### Participants

Women (*n* = 1500) were recruited at baseline (1998) from the Western Australian general population aged ≥ 70 years by mail using the electoral roll. Participants had an expected survival beyond 5 years, as part of the 5-year double-blind randomised controlled trial, the Calcium Intake Fracture Outcome Study (CAIFOS) [20]. A food frequency questionnaire (FFQ) was completed by 1485 women at baseline. Individuals with implausible energy intakes (< 2100 kJ or > 14,700 kJ, *n* = 17), missing any variable required to compute the frailty index (*n* = 73) or without information on smoking history (*n* = 5) were not included in the analysis. This resulted in a sample size of 1390 for the current study. Ethics approval was granted by the Human Research Ethics Committee of The University of Western Australia. The trial was retrospectively registered at the Australian New Zeland Clinical Trials Registry (#ACTRN12615000750583) and complied with the Declaration of Helsinki. Human ethics approval for use of data linkage was provided by the Western Australian Department of Health Human Research Ethics Committee (project #2009/24). Written, informed consent was obtained from each participant.

### Dietary assessment

A validated semi-quantitative FFQ (Cancer Council of Victoria, DQES V2.0) was used to assess dietary intake at baseline [21–23]. This FFQ assesses the usual frequency of food intake in the previous 12 months including 74 food items. An additional 27 food and alcoholic beverage items assessed using questions such as “how many different vegetables do you usually eat per day?” The FFQ calculates portion size by use of 3 photographs of scaled portions for 4 different commonly consumed food types. Nutrient intake calculations were analysed by Cancer Council Victoria primarily through the NUTTAB95 food nutrient database and supplemented by other data if required. This provided estimates for total protein, alcohol, calcium and energy intake. A research assistant supervised the completion of the questionnaire with food models, cups, spoons, and charts provided to enhance accuracy.

### Nitrate from plant sources

A comprehensive plant-based food reference nitrate database with nitrate values from 304 plant-based foods from 64 countries was used to calculate nitrate values of all plant-based foods (vegetables, fruits, cereals, herbs, spices, pulses and nuts) [24]. The nitrate content of plant foods differs according to country of cultivation; consequently, the following strategy was employed ; (i) median value for each plant food adopted if there were three or more entries in the database for Australia; (ii) median of values for all Oceania (Australia, New Zealand, and surrounding islands) was used if there were fewer than three entries in the database for Australia; (iii) the median of values for all countries in the database was used if there were fewer than three entries available for Oceania. The median nitrate value (mg/g) of each plant-based food was multiplied by the estimated quantity of the plant-based food consumed (g/d). The nitrate values of each individual food item were summed to obtain total plant nitrate based on the addition of nitrate derived from vegetables, beans, grains, and fruit per day. A detailed list of the food items in the aforementioned categories are in Supplementary Table 1.

### Animal-sourced nitrate intake

Red meat, dairy, seafood, and poultry were used for the calculation of naturally occurring animal-sourced nitrate intake. Meat with nitrate as an allowed additive (processed meat) was calculated separately due its link with detrimental health effects [25]. To calculate animal-sourced nitrate intake, a recently published nitrate reference database for animal-sourced food products, with data from 51 countries, was used [26]. To determine total animal-sourced nitrate consumed (mg/d), the amount of the specific animal-source food consumed (g/d) was multiplied by its median nitrate content (mg/g). Foods contributing to total animal nitrate is presented in Supplementary Table 1.

### Frailty index

Frailty at baseline was determined using a standard procedure described by Searle et al. [27] to create the FI. The proposed criteria requires between 30 and 40 variables across multiple health domains. These criteria included disability in activities of daily living (ADL), instrumental ADL, restricted activity, physical function (e.g., impaired walking, impaired grip strength) and general cognition, depression/mood, self-rated health and co-morbidity. In brief, our FI comprised of 33 items across health domains sourced from questionnaires including the Short form-36 and Barthel index, as well as objective measures of body composition, grip strength, timed-up-and-go performance, and blood pressure obtained at participants baseline clinical visit. Prevalent disease states (e.g. coronary heart disease, chronic heart failure, cerebrovascular disease, cancer, diabetes, arthritis and chronic lung disease) were determined using primary discharge diagnoses from hospital records over the previous 18-years (1980–1998) obtained from the Western Australian Data Linkage System (Department of Health Western Australia, East Perth, Australia) and the Western Australia Hospital Morbidity Data Collection. International Classification of Diseases (ICD) coded diagnosis data were used for each different disease states and pertained to all inpatient admissions (public and private) in Western Australia. Each variable was coded with a ‘1’ indicating presence of the health variable deficit or ‘0’ indicating absence of the health variable deficit. The total score across these variables were summed and divided by 33 to obtain the FI, scored from 0 to 1. Women were categorised as non-frail (FI < 0.25) or frail (FI ≥ 0.25) based on previous work [28]. A list of the 33 selected variables in this cohort and relevant scoring criteria is detailed previously [29], where we have also shown our FI to be linked with increased risk of hospitalised falls, fractures and mortality in this cohort.

### Further assessments

Women completed questionnaires regarding their smoking history, with anyone who had smoked more than one cigarette per day for more than three months at any point in their life classified as either a current smoker or an ex-smoker. Baseline venous blood samples (plasma) were collected in the morning (between 0830 and 1030 h) after an overnight fast and stored at -80 °C. Total plasma 25-hydroxyvitamin D (25OHD) concentration was determined at baseline in 1256 women using a validated LC-MS/MS (Liquid Chromatography Tandem Mass Spectrometry) method at the RDDT Laboratories (Bundoora, VIC, Australia) which measured plasma 25OHD_2_ and 25OHD_3_, as described previously [30]. Both 25OHD_2_ and 25OHD_3_ were summed to obtained total plasma 25OHD concentration. The coefficients of variation (CVs) were 10.1% at a mean concentration of 12 nmol/L for 25OHD_2_ and 11.3% at a mean concentration of 60 nmol/L for 25OHD_3_. Season when the blood sample was collected (Summer [December to February], Autumn [March to May], Winter [June to August], and Spring [September to November]) was combined into two groups for descriptive purposes, Summer/Autumn vs. Winter/Spring.

### Statistical analysis

Statistical analysis was performed using IBM SPSS Statistics for Windows (V24.0 IBM Corp., Armonk, NY, USA) and R (V3.4.2, R Foundation for Statistical Computing, Vienna, Austria). Generalised regression models were used to examine the cross-sectional association between nitrate intake (total plant and total animal nitrate, as separate exposures) and the FI expressed as a continuous variable for illustrative purposes only. P-values for the overall effect of the exposure on the outcomes (false discovery rate corrected) and for a test of non-linearity were obtained using likelihood ratio tests to compare appropriate nested models. Associations are presented graphically using the ‘effects’ R package [31]. For the primary analysis, we examined the cross-sectional association between nitrate intake (total plant and total animal nitrate, as separate exposures) and the presence of frailty. To allow associations to be non-linear, restricted cubic splines within logistic regression models were used to examine the relationship between nitrate intake and the presence of frailty using the ‘rms’ R package [32]. Odds ratio (OR) estimates were relative to a reference value being the median nitrate intake of participants in Q1 and were plotted against the respective outcomes with 95% confidence bands provided. Wald tests were used to obtained p-values. For visual simplicity only, the x-axis was truncated at 3 SD above the mean. For all analysis, two models of adjustment were adopted including an age-adjusted (Model 1) and multivariable-adjusted model (Model 2) including age, smoking history, protein and alcohol as well as energy intake (less energy contribution from protein and alcohol). As the FI includes various comorbidities (e.g., prevalent cardiovascular disease, diabetes, arthritis) as well as physical activity and body composition measures (e.g., BMI), these were not added to Model 2.

### Additional analysis

To examine the robustness of the relationship between nitrate and the presence of frailty, additional covariates, with the potential to influence this relationship were added to Model 2. This included, dietary calcium, vitamin C, fibre, vitamin E [33] as well as two measures of overall diet quality: (i) Nutrient Rich Food Index (NRFI) per 1000 kJ [34]; and (ii) a diet adherence measure to the 2013 Australian Dietary Guidelines (ADG) [35]. Further, as lower circulating 25OHD has been linked with greater frailty risk [36], plasma 25OHD (and season of sample collection) were added to Model 2. As we demonstrated that total plant nitrate was linked with lower odds for frailty, we examined this further by considering the different contributing groups to total plant nitrate, including nitrate derived from vegetables, beans, grains and fruit, separately. Finally, as green leafy vegetables, comprising lettuce, spinach and celery represent a major source of plant nitrate in this cohort [14]), we examined the relationship between total green leafy vegetable intake (substituted for plant nitrate) and the presence of frailty in our multivariable-adjusted model.

## Results

Baseline characteristics according to quartiles of plant- or animal-derived nitrate per day are presented in Table 1 and Supplementary Table 2, respectively. Median (IQR) daily total plant and total animal nitrate was 72.1 (55.6–90.0) mg/d and 3.5 (2.2–5.1) mg/d, respectively. When considering plant nitrate, those with highest intakes (Q4) were found to have greater energy and protein consumption, as well as higher circulating 25OHD concentrations, compared to those with the lowest intake (Q1). When stratified by quartiles of animal nitrate intake, those within the highest quartile of intake (Q4) were slightly younger and had greater consumption of protein, alcohol and plant nitrate compared to those with the lowest intake (Q1).

**Table 1:** Baseline characteristics in all participants by quartiles of plant nitrate intake^1^

	All Participants	Quartiles of plant nitrate intake				*p*-value
		Quartile 1 < 55.6 mg/d	Quartile 2 55.6 to < 72.1 mg/d	Quartile 3 72.1 to < 90.0 mg/d	Quartile 4 ≥ 90.0 mg/d	
**Number**	1390	348	347	348	347	
Age, years	75.1 ± 2.7	75.2 ± 2.8	75.0 ± 2.7	75.1 ± 2.7	75.3 ± 2.6	0.416
Body mass index (BMI), kg/m^2^	27.1 ± 4.7	26.9 ± 4.6	27.5 ± 4.6	27.0 ± 4.8	27.3 ± 4.8	0.346
Smoked ever, yes n (%)	519 (37.3)	132 (37.9)	121 (34.9)	126 (36.2)	140 (40.3)	0.478
Total energy intake (kJ/d)	7119 ± 2071	6050 ± 1606	7010 ± 1894	8024 ± 2236	8024 ± 2236	< 0.001
Alcohol intake, g/d	1.9 (0.3–9.9)	1.7 (0.2–9.6)	1.6 (2.6–9.2)	1.9 (0.4–10.4)	2.3 (0.3–10.2)	0.785
Protein intake, g/d	79.7 ± 26.3	65.2 ± 19.9	77.3 ± 23.4	84.2 ± 24.6	92.3 ± 28.6	< 0.001
Energy (kJ/d) less protein + alcohol	5961 ± 1756	5142 ± 1435	5888 ± 1621	6150 ± 1688	6666 ± 1900	< 0.001
25OHD, mmol/L^2^	67.0 ± 28.1	63.3 ± 27.5	68.0 ± 29.1	67.8 ± 28.4	69.2 ± 27.0	0.043
Blood sample collection season^2^						
*Summer/Autumn, n (%)*	314 (22.6)	77 (23.9)	74 (23.9)	69 (22.5)	94 (29.5)	0.190
*Winter/Spring, n (%)*	942 (67.8)	245 (76.1)	235 (76.1)	237 (77.5)	225 (70.5)	
Plant nitrate intake, mg/d	72.1 (55.6–90.0)	44.7 (37.1–50.5)	63.5 (60.0-68.1)	80.2 (75.7–84.9)	104.8 (96.1-122.3)	< 0.001
Animal nitate intake, mg/d	3.5 (2.2–5.1)	2.8 (1.8–4.1)	3.6 (2.3–5.1)	3.7 (2.3–5.2)	4.2 (2.6–5.4)	< 0.001

^1^ Data presented mean ± SD, median (inter quartile range) or n (%) where appropriate. ^2^*n*=1256. One-way ANOVA, Kruskall-Wallis test or Pearsons Chi-square test used to examine for differences across quartiles of plant nitrate intake where appropriate.

**Fig. 1:**
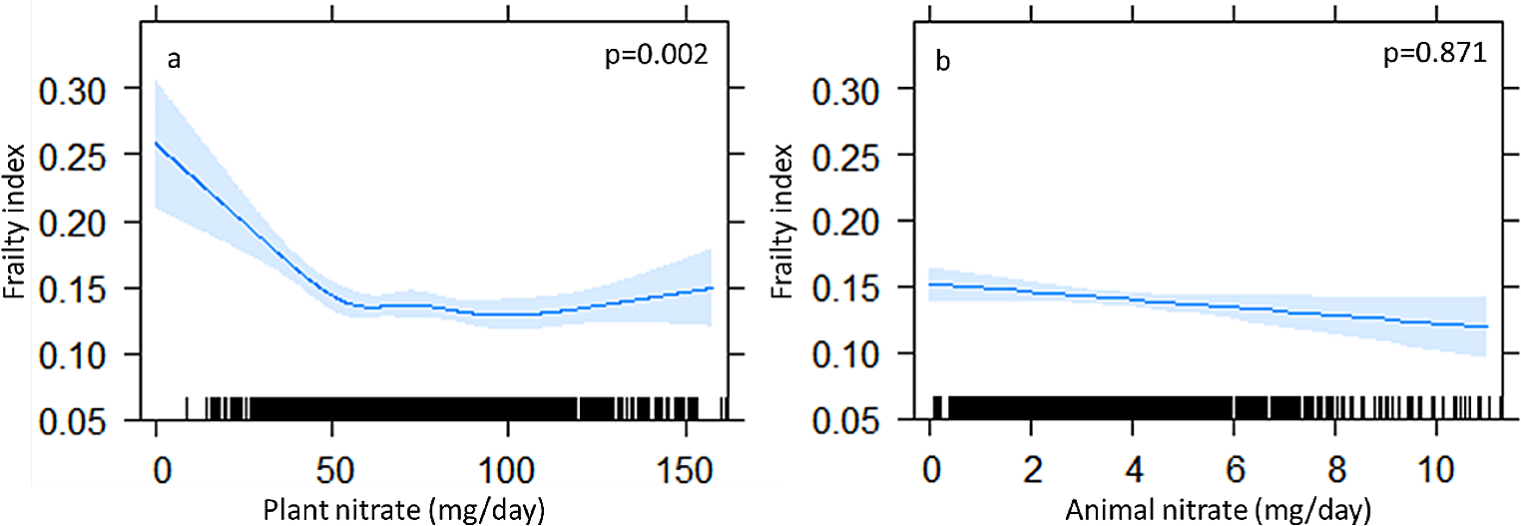
Multivariable-adjusted relationship between dietary nitrate derived from (**a**) plants and (**b**) animals with the frailty index obtained by generalized regression models. Shading represents 95% confidence intervals. The rug plot along the bottom of each graph depicts each observation. Models adjusted for age, smoking history, energy intake, protein and alcohol intake (Model 2).

**Fig. 2:**
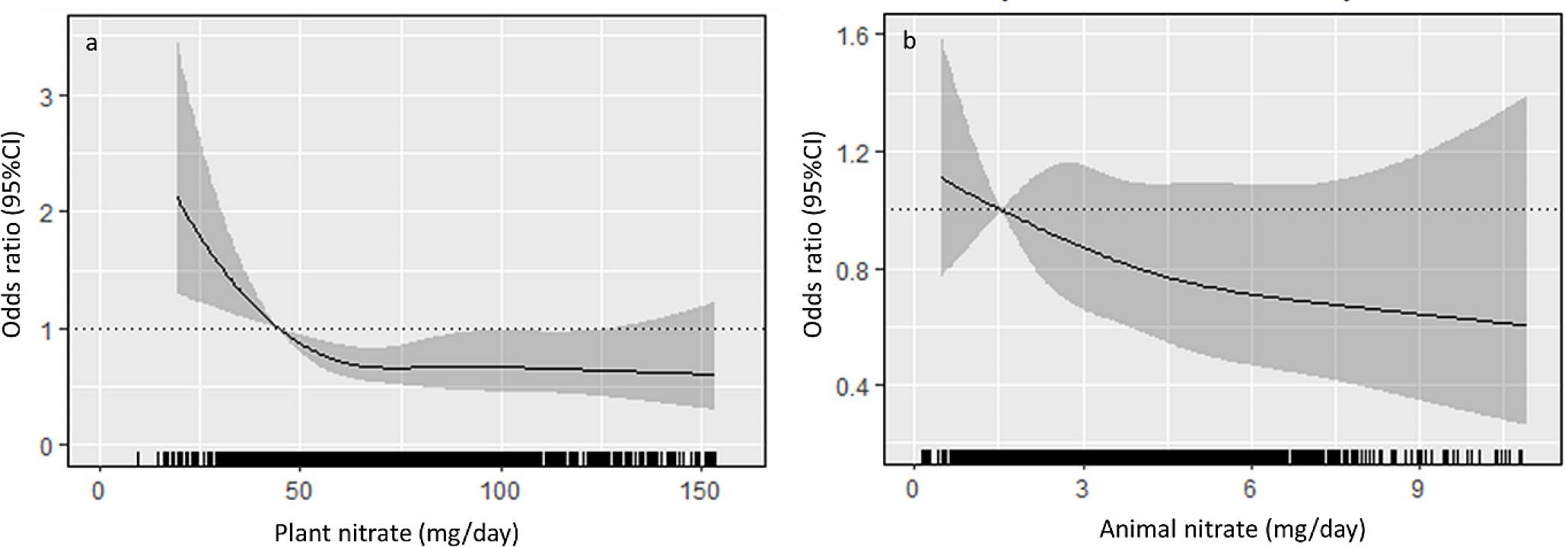
Odds ratios from multivariable-adjusted logistic regression models with restricted cubic spline curves describing the association between nitrate derived from plant (**a**) and animal sources (**b**) with the presence of frailty. Odds ratios are based on models adjusted for age, smoking history, energy, protein and alcohol intake (Model 2). The odds ratio compares the specific intake of nitrate (horizontal axis) to the median intake in the lowest quartile (44.7 mg/d for plant nitrate and 1.6 mg/d for animal nitrate). Shading represents 95% confidence regions. The rug plot along the bottom of each graph depicts an observation

A diagrammatic representation of the non-linear relationship (p for non-linearity < 0.001) between plant nitrate and the FI is presented in Fig. 1a. Specifically, an inverse association between plant nitrate and the FI was observed with a nadir recorded once an intake of approximately > 55 mg/d was attained (lower bound for Q2). A non-linear relationship (p for non-linearity = 0.028) was also observed between higher total plant nitrate (Q2, Q3, Q4) and the presence of frailty with a nadir recorded once an intake of approximately > 64 mg/d was attained. (Fig. 2a). Compared to women with the lowest nitrate intake (Q1), women in Q2 to Q4 had 31%, 33% and 34% lower odds for frailty, respectively (all *p* < 0.05, Table 2). Total animal nitrate intake was not associated with the FI (Fig. 1b) nor the presence of frailty (Fig. 2b; Table 2). A diagrammatic representation for the non-linear relationship (p for non-linearity < 0.001) between total nitrate intake and the FI is found in Supplementary Fig. 1, with a nadir recorded once an intake of approximately ~ 60 mg/d was attained (lower bound for Q2). Compared to women with the lowest total nitrate intake (Q1), women in Q2, Q3 and Q4 had 32%, 34% and 35% lower odds for the presence of frailty, respectively (Supplementary Table 3, Supplementary Fig. 2).

**Table 2:** Odds ratio (95%CI) for the presence of frailty by quartiles of dietary nitrate intake from plant and animal sources

		Nitrate intake^1^			
		Quartile 1 < 55.6 mg/d	Quartile 2 55.6 to < 72.1 mg/d	Quartile 3 72.1 to < 90.0 mg/d	Quartile 4 ≥ 90.0 mg/d
***Plant-nitrate***	*Frailty, n (%)*	85 (24.4)	65 (18.7)	67 (19.3)	59 (17.0)
	*Model 1*	Ref.	**0.69 (0.57–0.83)**	**0.66 (0.50–0.88)**	**0.68 (0.48–0.97)**
	*Model 2*	Ref.	**0.69 (0.56–0.84)**	**0.67 (0.50–0.90)**	**0.66 (0.45–0.98)**
***Animal-nitrate***		**Quartile 1** < 2.2 mg/d	**Quartile 2** 2.2 to < 3.5 mg/d	**Quartile 3** 3.5 to < 5.1 mg/d	**Quartile 4** ≥ 5.1 mg/d
	*Events, n (%)*	77 (2.8)	71 (20.2)	66 (19.1)	62 (17.5)
	*Model 1*	Ref.	0.88 (0.68–1.13)	0.80 (0.60–1.05)	0.75 (0.53–1.06)
	*Model 2*	Ref.	0.88 (0.67–1.16)	0.78 (0.57–1.09)	0.71 (0.46–1.08)

^1^Estimated odds and 95%CI from logistic regression analysis comparing the median nitrate intake from each quartile (Q) compared to Q1. Median intake Q1, Q2, Q3 and Q4 for plant-derived nitrate was 44.7, 63.5, 80.2 and 104.8 mg/d, respectively. Median intake Q1, Q2, Q3 and Q4 for animal-derived nitrate was 1.6, 2.9, 4.2 and 6.1 mg/d, respectively. Model 1: adjusted for age. Model 2: age + smoking history, energy intake, protein and alcohol intake. Bolded indicates *p* < 0.05 compared to Q1

### Additional analysis

The addition of dietary calcium, vitamin C or two different indices of diet quality (ADG or NRFI per 1000 kJ) to Model 2 did not appear to influence the relationship between plant nitrate and the presence of frailty (Supplementary Table 4). When plasma 25OHD was added to Model 2, results remained unchanged. Specifically, women in Q2, Q3 and Q4 of total plant nitrate had 30%, 35% and 39% lower odds for frailty, respectively (Supplementary Table 4). However, when dietary vitamin E was added to Model 2, only women in Q2 and Q3 (both *p* < 0.05), but not Q4 of total plant nitrate, had 27% lower odds for frailty. Similarly, when dietary fibre intake was added to Model 2, only women in Q2 and Q3 (both *p* < 0.05), but not Q4 of total plant nitrate, had 28% lower odds for frailty. When considering the different sources of total plant nitrate, the leading contributors were vegetable (65.5%), fruit (20.3%), grains (7.2%) and beans (7.0%). Notably, the median nitrate provided from vegetables (46.6 [ 34.2–62.6] mg/d) was also substantially higher compared to beans (4.1 [ 2.1-7.0] mg/d), fruit (13.2 [8.6–18.7] mg/d) and grain (4.3 [ 3.2-6.0] mg/d). Adopting Model 2, higher nitrate from vegetables (Q2, Q3, Q4, ~ 41%), beans (only Q2 27%) and grains (Q3 28%, Q4 36%) were associated with lower odds for frailty (Supplementary Table 5, Supplementary Fig. 3). In our multivariable-adjusted analysis where plant nitrate was removed and substituted with its major source being green leafy vegetables, women with higher intakes in Q2 (median 13 g/d; OR 0.68 95%CI 0.55–0.83), Q3 (median 21 g/d; OR 0.66 95%CI 0.49–0.90) and Q4 (median 32 g/d; OR 0.65 95%CI 0.44–0.97) had lower odds for frailty, compared to those with the lowest intake (Q1 median 6 g/d) (Supplementary Fig. 4).

## Discussion

The main finding from this study was that higher total plant nitrate intake, largely driven by consumption of vegetables and grains, was associated with lower odds for frailty in community-dwelling older Australian women. This is the first study, to our knowledge, which reports an association between habitual nitrate intake and frailty. Specifically, women with higher plant nitrate intakes (Q2-Q4) were 31–34% less likely to be frail when compared with women with the lowest (Q1) plant nitrate intake. There did not appear to be any additional benefit once plant-nitrate intakes of ~ 64 mg/day were attained. No association was found between total animal nitrate intake and frailty.

The mechanisms through which dietary nitrate impacts frailty are likely to be multifactorial, and consequent to a nitrate-related increase in the bioavailability of NO [10, 11]. NO has pleiotropic actions in the human body and is implicated in the function of a range of different body systems. Namely, as a potent neurotransmitter and modulator of cerebral blood flow, NO exerts effects on cognitive function [19, 37]. NO also impacts muscle function and metabolism, as increased NO bioavailability consequent to nitrate intake has been linked to increased efficiency of mitochondrial respiration, alterations to muscle Ca^2+^ handling, a reduced ATP cost of muscle force production, and increased blood flow to the muscle [14, 18, 38, 39]. Dietary nitrate intake has also been shown to modify aspects of cardiovascular health, reducing endothelial dysfunction, vascular stiffness and blood pressure [9, 34, 40, 41]. Most importantly, in the current cohort, dietary nitrate intake (especially from plants) is inversely related with muscle function measures (e.g., grip strength, timed-up-and go) [14] and atherosclerotic cardiovascular disease [34]. Considering this captures key components of the FI, it provides evidence for the underlying mechanisms to support our results. Given that higher nitrate intakes have been reported in other work to benefit these components of frailty [9, 13, 15, 42] , including cognitive health [43, 44], this relationship likely reflects an accumulation of the aforementioned multi-system benefits.

Our data suggests that an intake of > 64 mg/d of plant-derived nitrate, largely from consumption of vegetables and grains, may be sufficient to maximise beneficial associations with frailty, with no additional reductions in the likelihood of frailty observed at higher intakes. In contrast, acute and short-term studies exploring the impact of nitrate on health markers have typically suggested much higher doses of nitrate are required to maximise (or even elicit) beneficial effects. For example, a meta-analyses investigating the effects of acute dietary nitrate intake on various markers of cardiovascular risk factors recommended that a 130–259 mg bolus dose of nitrate was required to reduce systolic blood pressure [45]. In another meta-analysis reporting the acute positive effects of dietary nitrate on muscle power, a range of 400–985 mg of nitrate, mostly obtained from beetroot juice, was provided across all the studies [13]. However, recent evidence has suggested that when ingested habitually, dietary nitrate intake of 100–200 mg/day may be more effective for reducing systolic blood pressure than substantially higher intakes (400 mg/day) [46]. It is currently unclear why this discrepancy in optimal dose is present. However, Babateen et al., suggested that high doses of nitrate over prolonged periods may result in the development of tachyphylaxis, resulting in the downregulation of vascular eNOS activity, and decreased NO production via oxidation of L-arginine [46, 47]. We speculate that similar mechanisms may be the reason for the nadir observed in the current study, and as such any dietary nitrate intake above that of ∼ 64 mg would have no additional benefit in reducing the likelihood of frailty. Alternatively, it is possible that low doses of nitrate in the habitual diet allow the accrual of small, incremental benefits over time which may not be detectable in acute or short-term studies against background measurement error.

No association was recorded between dietary nitrate intake from animal sources and frailty. Notwithstanding the prospect that animal nitrate truly has no influence on frailty, there may be various explanations for this finding in the current study. Firstly, assuming that dietary nitrate from both plant and animal sources exerts a similar effect per mg, nitrate intake from animal sources may not be sufficient to affect frailty. For example, median (IQR) daily total plant and total animal nitrate was 72.1 (55.6–90.0) mg/d and 3.5 (2.2–5.1) mg/day, respectively. Therefore, it may be considerably harder to establish any influence of animal nitrate without a larger range of intakes, especially considering intake from plant sources is always likely to confound results. Of course, it is possible that plant nitrate intake also has a stronger association with frailty compared to animal sources due to the food matrixes that are inherent to plant-based sources of nitrate which have consistently been shown to be beneficial for overall health [48–51]. Plant-based sources of nitrate also contain other active components such as polyphenols and other antioxidants that might lead to (i) a greater NO yield per mg of nitrate, and (ii) prolonged NO bioactivity by scavenging superoxide and other free-radicals [52–54]. Of note, greater effects of plant-based nitrate on blood pressure, compared to an isolated supplement of sodium nitrate (an allowed additive for processed meats), have previously been observed [55]. Consuming higher amounts of ultra processed foods, which include processed meats, has also been shown to increase risk of incident frailty, assessed using the Fried frailty phenotype, in a prospective cohort of older Spanish adults over 3.5 years (*n* = 1822, ≥ 60 years, 51.3% female) [56]. Similarly, when considering physically frail middle-aged adults from the UK Biobank (*n* = 19,913, 58 years, 59.4% female), frequent consumption of processed meat was also associated with increased mortality risk [57].

From a public health perspective, the relatively low dose of dietary nitrate (> 64 mg/day) that may be required to reduce the likelihood of frailty in older community-dwelling women is encouraging. To put these findings into perspective, just one serving of green leafy vegetables, or approximately 50 g of beetroot, would provide 75 mg of nitrate. Alternatively, individuals with low nitrate intakes (e.g., 35 mg/day, median of Q1), consuming approximately half a serve extra per day of green leafy vegetables would ensure a nitrate intake of more than 64 mg. Potential benefits are supported in our analysis substituting plant nitrate with green leafy vegetable intake that demonstrated approximately 35% lower odds for frailty when as little as 1 cup of green leafy vegetables (e.g., ~ 20–30 g of spinach) was consumed. Using vegetables as a means of increasing dietary nitrate is well-accepted amongst older adults with very high compliance (97–98%) in studies lasting 4–5 weeks where participants were required to consume > 200 mg/day [58, 59]. Nitrate-rich vegetables are also relatively inexpensive and easily accessible. Indeed, in 2023, 64 mg of nitrate could be obtained from commercially available vegetables costing as little as 0.08 GBP (USD 0.10) [60]. Given that increased dietary nitrate intake has been linked to multiple domains of frailty such as cardiovascular health, muscle strength, and cognitive function, older individuals may be recommended to increase their intake of nitrate-rich vegetables as a means of reducing the likelihood of frailty [12]. Although increasing nitrate intake above this threshold is not likely to reduce frailty odds further, diets rich in vegetables are generally considered to be healthier [5] (e.g., due to the provision of fibre and other micronutrients implicated in various aspects of human health), and hence, 1–2 serving of green-leafy vegetables should not be considered an upper limit, but rather a lower-bound.

The recruitment of a large cohort of Australian community-dwelling older women that were representative of their demographic is a major strength of this study. Whilst this is the first study to provide evidence for an inverse non-linear association between plant-derived dietary nitrate and frailty assessed using the validated cumulative deficits model [27], there are several limitations to the work. For example, the data are observational and cross-sectional in nature, therefore causality cannot be established. Future studies should examine this association in a longitudinal design, to establish whether nitrate intake is associated with changes in frailty. To minimise the potential for residual confounding, we included a range of dietary covariates including protein, calcium, vitamin C, fibre and vitamin E intake, as well as two measures of overall diet quality (NRFI as well as ADG) and still report comparable results. However, the addition of vitamin E and fibre to the multivariable-adjusted model recorded significantly lower odds for frailty only in individuals in Q2 and Q3 (but not Q4) of plant nitrate intake, when compared to the Q1. As vegetables are a rich source of nitrate, vitamin E and fibre, this may have influenced our results. Alternatively, it should be acknowledged that there are other components in vegetables that could be contributing to the observed associations. Additionally, although nitrate intake was estimated from validated food databases for the current study, some error may be introduced due to variations in cooking methods [61, 62] or growing conditions of the plants [63, 64], so that actual nitrate intake could vary. To minimise error, we adopted recently compiled nitrate databases which also took cooking method into account.

In conclusion, this investigation highlights the potential detrimental impact of low plant nitrate intakes for the likelihood of frailty in community dwelling older women. Notably, small increases in intakes of nitrate derived from both vegetables and grains appear most beneficial. Public health messaging should continue to promote 5–6 serves per day of vegetables, whilst highlighting the importance of including at least one serve per day of nitrate-rich vegetables (e.g., spinach, beetroot, rocket, pak choi). Habitual consumption of such vegetables (75 g per day) would provide sufficient dietary nitrate to obtain levels typically linked to a range of health benefits [12]. Finally, further work with longitudinal research designs is needed to support these findings before population-specific recommendations may be considered.

## Electronic supplementary material

Below is the link to the electronic supplementary material.

Supplementary Material 1
