# Supplementary material for: Higher plant-derived nitrate intake is associated with lower odds of frailty in a cross-sectional study of community-dwelling older women
Source: Eur J Nutr. 2024 May 18;63(6):2281–90. doi: 10.1007/s00394-024-03412-z (PMC11377636; doi:10.1007/s00394-024-03412-z)
Supplement: Supplementary file 1 — Supplementary Material 1 [file 394_2024_3412_MOESM1_ESM.docx]

**Supplementary Table 1.** Foods used to as part of the different categories on nitrate intake.

| **Total plant nitrate** | **Vegetable** | Potatoes, Tomato sauce, Tomatoes, Capsicum, Lettuce, Cucumber, Celery, Beetroot, Carrots, Cabbage, Cauliflower, Broccoli, Spinach, Peas, Pumpkin, Onion, Garlic, Mushrooms, Zucchini, Avocado |
| --- | --- | --- |
|  | **Beans** | Green beans, Sprouts, Baked beans, Tofu, Other beans |
|  | **Grains** | High fibre bread, White bread, Wholemeal bread, Rye bread, Multigrain bread, All-bran cereal, Other bran cereal, Weetabix cereal, Cornflakes cereal, Porridge, Muesli, Rice, Pasta |
|  | **Fruit** | Tinned fruit, Fruit juice, Oranges, Other citrus fruits, Apples, Pears, Bananas, Melons, Pineapple, Strawberries, Apricots, Peaches, Mango |
| **Total animal nitrate** | **Animal products** | Full cream milk, Reduced fat milk, Skimmed milk, Butter, Eggs, Hard cheese, Firm cheese, Soft cheese, Ricotta cheese, Cream cheese, Low fat cheese, Flavoured milk, Ice cream, Yoghurt, Beef, Veal, Chicken, Lamb, Pork, Grilled fish, Fried fish, Tinned fish |
|  | **Processed meats** | Sausages, Bacon, Ham, Salami |

**Supplementary Table 2.** Baseline characteristics in all participants by quartiles of animal nitrate intake^1^.

|  | **All Participants** | **Quartiles of animal nitrate intake** | | | |  |
| --- | --- | --- | --- | --- | --- | --- |
|  |  | **Quartile 1**  <2.2 mg/d | **Quartile 2**  2.2 to <3.5 mg/d | **Quartile 3**  3.5 to <5.1 mg/d | **Quartile 4**  ≥5.1 mg/d | **p-value** |
| **Number** | 1390 | 338 | 351 | 346 | 355 |  |
| Age, years | 75.1 ± 2.7 | 75.5 ± 2.8 | 75.1 ± 2.7 | 75.1 ± 2.6 | 74.9 ± 2.7 | 0.045 |
| Body mass index (BMI), kg/m^2^ | 27.1 ± 4.7 | 26.8 ± 4.8 | 27.3 ± 4.9 | 27.1 ± 4.6 | 27.4 ± 4.6 | 0.267 |
| Smoked ever, yes n (%) | 519 (37.3) | 131 (38.8) | 123 (35.0) | 127 (36.7) | 138 (38.9) | 0.532 |
| Energy intake(kJ/d) less protein and alcohol | 5961 ± 1756 | 4868± 1217 | 5796 ± 1701 | 6126 ± 1543 | 7008 ± 1793 | <0.001 |
| Alcohol intake, g/d | 1.9 (0.3-9.9) | 0.9 (0.0-8.8) | 1.7 (0.3-9.7) | 2.6 (0.3-10.4) | 2.8 (0.4-10.8) | 0.004 |
| Protein intake, g/d | 79.7 ± 26.3 | 58.9 ± 15.4 | 73.9 ± 18.3 | 83.6 ± 20.2 | 101.5 ± 28.6 | <0.001 |
| 25OHD, mmol/L^2^ | 67.0 ± 28.1 | 66.2 ± 28.7 | 67.2 ± 28.1 | 66.3 ± 26.2 | 68.5 ± 29.2 | 0.873 |
| Blood sample collection season^2^ |  |  |  |  |  |  |
| *Summer/Autumn, n (%)* | 314 (22.6) | 89 (28.3) | 76 (24.2) | 69 (22.0) | 80 (25.5) | 0.499 |
| *Winter/Spring, n (%)* | 942 (67.8) | 225 (71.7) | 238 (75.8) | 245 (78.0) | 234 (74.5) |  |
| Plant nitrate intake, mg/d | 72.1 (55.6-90.0) | 63.1 (46.2-82.1) | 69.8 (53.7-89.0) | 73.8 (57.2-92.1) | 79.2 (63.0-96.2) | <0.001 |
| Animal nitrate intake, mg/d | 3.5 (2.2-5.1) | 1.5 (1.1-1.9) | 2.8 (2.4-3.2) | 4.2 (3.9-4.6) | 6.1 (5.4-7.1) | <0.001 |

^1^ Data presented mean ± SD, median (inter quartile range) or n (%) where appropriate. ^2^n=1256. One-way ANOVA, Kruskall-Wallis test or Pearsons Chi-square test used to examine for differences across quartiles of animal nitrate intake where appropriate.

**Supplementary Table 3.** Odds ratio (95%CI) for the presence of frailty by quartiles of total dietary nitrate intake from both plant and animal sources.

|  |  | **Nitrate intake^1^** | | | |
| --- | --- | --- | --- | --- | --- |
|  |  | **Quartile 1**  <59.8 mg/d | **Quartile 2**  59.8 to <76.4 mg/d | **Quartile 3**  76.4 to 94.4 mg/d | **Quartile 4**  ≥94.9 mg/d |
|  | *Events, n (%)* | 89 (25.6) | 63 (18.1) | 67 (19.4) | 57 (16.4) |
| ***Total nitrate*** | *Model 1* | Ref. | **0.67 (0.55-0.82)** | **0.66 (0.50-0.87)** | **0.67 (0.47-0.95)** |
|  | *Model 2* | Ref. | **0.68 (0.55-0.83)** | **0.66 (0.49-0.90)** | **0.65 (0.44-0.97)** |

^1^Estimated odds and 95%CI from logistic regression analysis comparing the median total nitrate intake from each quartile (Q) compared to Q1. Median intake Q1, Q2, Q3 and Q4 for total nitrate was 48.3, 69.0, 84.8 and 110.3 mg/d, respectively. Model 1: adjusted for age. Model 2: Model 1 + smoking history, energy intake, protein and alcohol intake. Bolded indicates p<0.05 compared to Q1.

**Supplementary Table 4.** Multivariable-adjusted odds ratios (95%CI) for the presence of frailty by quartiles of plant-derived nitrate intake after individually adjusting for intakes of calcium, vitamin C, vitamin E and fibre as well as diet quality using the Nutrient Rich Food Index (NRFI, standardised per 1000 kJ of energy intake), Australian Dietary Guideline Index (ADG) and circulating plasma 25-hydroxyvitamin D (25OHD).

|  | **Plant-derived nitrate ^1^** | | | |
| --- | --- | --- | --- | --- |
|  | **Quartile 1**  <55.6 mg/d | **Quartile 2**  55.6 to <72.1 mg/d | **Quartile 3**  72.1 to <90.0 mg/d | **Quartile 4**  ≥90.0 mg/d |
| *Frailty, n (%)* | 85 (24.4) | 65 (18.7) | 67 (19.3) | 59 (17.0) |
| *Model 2* | Ref. | **0.69 (0.56-0.84)** | **0.67 (0.50-0.90)** | **0.66 (0.45-0.98)** |
| *Model 2 + calcium* | Ref. | **0.69 (0.57-0.85)** | **0.67 (0.50-0.90)** | **0.66 (0.45-0.97)** |
| *Model 2 + vitamin C* | Ref. | **0.68 (0.55-0.83)** | **0.65 (0.48-0.89)** | **0.64 (0.42-0.96)** |
| *Model 2 + vitamin E* | Ref. | **0.73 (0.59-0.89)** | **0.73 (0.54-1.00)** | 0.77 (0.52-1.15) |
| *Model 2 + fibre* | Ref. | **0.72 (0.58 -0.89)** | **0.72 (0.52 -1.00)** | 0.75 (0.49 -1.16) |
| *Model 2 + NRFI per 1000 kJ^2^* | Ref. | **0.69 (0.57-0.83)** | **0.67 (0.50-0.88)** | **0.69 (0.48-0.99)** |
| *Model 2 + ADG^2^* | Ref. | **0.68 (0.55-0.83)** | **0.65 (0.48-0.88)** | **0.66 (0.44-0.97)** |
| *Model 2 + 25OHD and season^3^* | Ref. | **0.70 (0.57-0.87)** | **0.65 (0.47-0.88)** | **0.61 (0.41-0.91)** |

^1^Estimated odds and 95%CI from logistic regression models comparing the median plant nitrate intake from each quartile (Q) compared to Q1. Median plant nitrate in Q1, Q2, Q3 and Q4 was 44.7, 63.5, 80.2 and 104.8 mg/d, respectively. Bolded indicates p<0.05 compared to Q1. ^2^ energy, protein and alcohol intake not included in this model as the diet indices also consider these aspects. ^3^n=1256

**Supplementary Table 5.** Odds ratio (95%CI) for the presence of frailty by quartiles of dietary nitrate intake from a variety of plant sources.

|  |  | **Sources of nitrate ^1^** | | | |
| --- | --- | --- | --- | --- | --- |
|  |  | **Quartile 1** | **Quartile 2** | **Quartile 3** | **Quartile 4** |
| *Vegetable nitrate* | *Frailty, n (%)* | 91 (26.2) | 63 (18.1) | 60 (17.3) | 62 (17.8) |
|  | *Model 2* | Ref. | **0.60 (0.49-0.75)** | **0.57 (0.43-0.76)** | **0.59 (0.41-0.87)** |
| *Bean nitrate* | *Frailty, n (%)* | 82 (23.6) | 58 (16.7) | 58 (16.7) | 78 (22.4) |
|  | *Model 2* | Ref. | **0.73 (0.56-0.95)** | 0.80 (0.60-1.06) | 0.95 (0.67-1.34) |
| *Fruit nitrate* | *Frailty, n (%)* | 77 (22.2) | 70 (20.1) | 53 (15.2) | 76 (21.9) |
|  | *Model 2* | Ref. | 0.81 (0.62-1.06) | 0.76 (0.57-1.03) | 0.84 (0.58-1.20) |
| *Grain nitrate* | *Frailty, n (%)* | 84 (24.2) | 68 (19.5) | 64 (18.4) | 60 (17.3) |
|  | *Model 2* | Ref. | 0.89 (0.72-1.09) | **0.72 (0.54-0.96)** | **0.64 (0.41-0.98)** |

^1^Estimated odds and 95%CI from logistic regression analysis comparing the median nitrate intake from each quartile (Q) compared to Q1. Median vegetable-derived nitrate in Q1, Q2, Q3 and Q4 was 25.0, 40.9, 53.7 and 75.8 mg/d, respectively. Median fruit-derived nitrate in Q1, Q2, Q3 and Q4 was 5.6, 10.8, 16.0 and 24.5 mg/d, respectively. Median bean-derived nitrate in Q1, Q2, Q3 and Q4 was 1.2, 2.9, 5.2 and 9.4 mg/d, respectively. Median grain-derived nitrate in Q1, Q2, Q3 and Q4 was 2.6, 3.8, 5.0 and 7.4 mg/d, respectively. Model adjusted for age, smoking history, energy, protein and alcohol intake (Model 2). Bolded indicates p<0.05 compared to Q1.


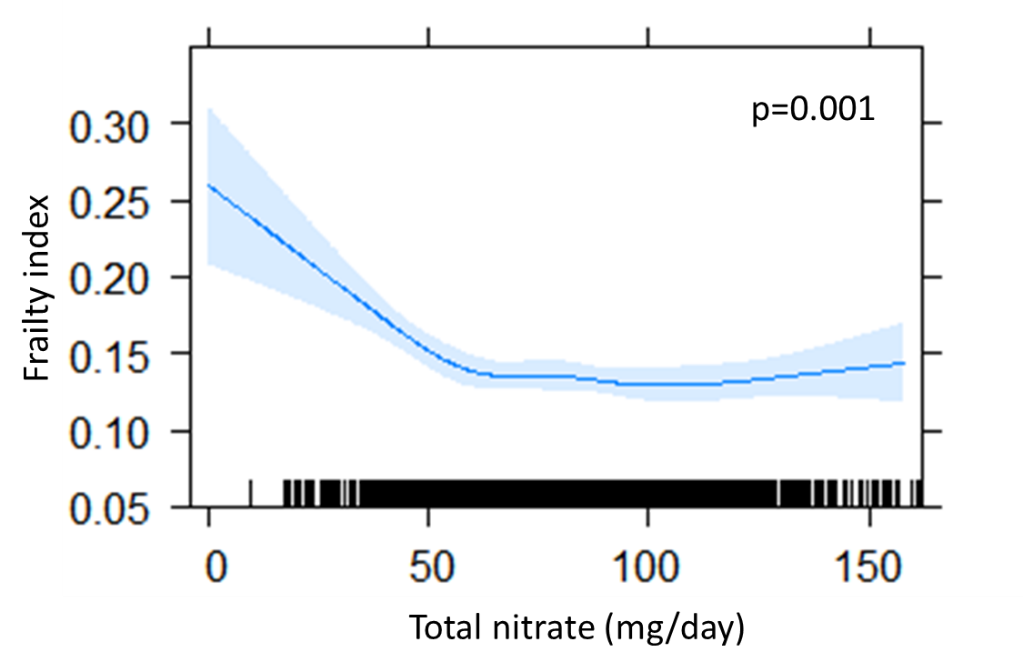


**Supplementary Figure 1.** Multivariable-adjusted relationship between total dietary nitrate derived from both plants and animals with the frailty index obtained by generalized regression models. Shading represents 95% confidence intervals. The rug plot along the bottom of each graph depicts each observation. Models adjusted for age, smoking history, energy intake, protein and alcohol intake (Model 2).


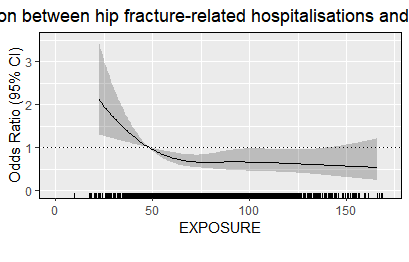


p=0.002

Total nitrate intake mg/d

**Supplementary Figure 2.** Odds ratios from multivariable-adjusted logistic regression models with restricted cubic spline curves describing the association between total nitrate intake with the presence of frailty. The odds ratio compares the specific intake of nitrate (horizontal axis) to the median intake in the lowest quartile. Shading represents 95% confidence regions. The rug plot along the bottom of each graph depicts an observation.

**
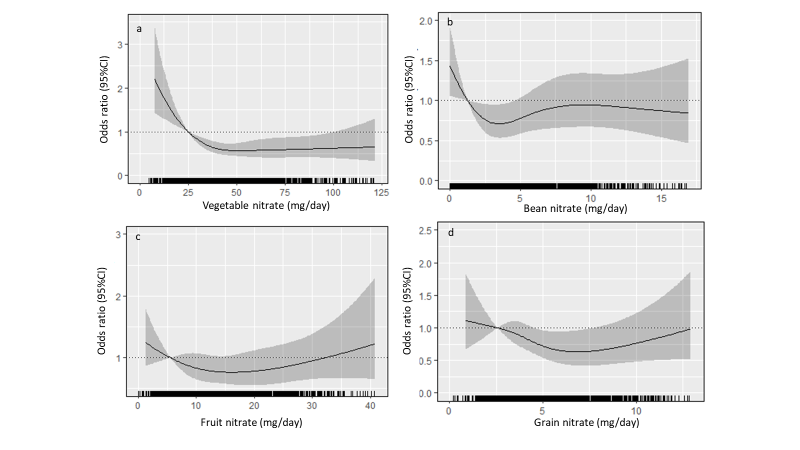
**

**Supplementary Figure 3.** Odds ratios from multivariable-adjusted logistic regression models with restricted cubic spline curves describing the association between nitrate derived from different plant sources including vegetables (a), beans (b), fruit (c) and grains (d) with the presence of frailty. Odds ratios are based on models adjusted for age, smoking history, energy, protein and alcohol intake (Model 2). The odds ratio compares the specific intake of nitrate (horizontal axis) to the median intake in the lowest quartile (see Supplementary Table 5). Shading represents 95% confidence regions. The rug plot along the bottom of each graph depicts an observation.


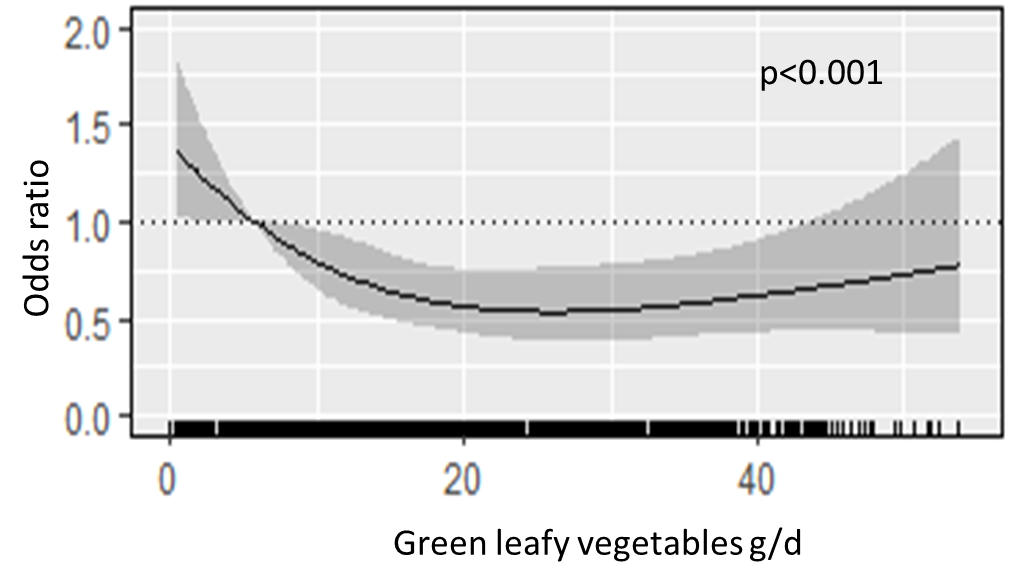


**Supplementary Figure 4.** Odds ratios from multivariable-adjusted logistic regression models with restricted cubic spline curves describing the association between green leafy vegetable intake with the presence of frailty. Odds ratios are based on models adjusted for age, smoking history, energy, protein and alcohol intake (Model 2). The odds ratio compares the specific intake of green leafy vegetable (horizontal axis) to the median intake in the lowest quartile (6 g/d). Shading represents 95% confidence regions. The rug plot along the bottom of each graph depicts an observation.
